# Editorial: Biological aspects of bone marrow failure

**DOI:** 10.3389/fonc.2023.1295823

**Published:** 2023-10-04

**Authors:** Panagiotis Diamantopoulos, Serine Avagyan, Elena Solomou

**Affiliations:** ^1^ First Department of Internal Medicine, Laiko General Hospital, National and Kapodistrian University of Athens, Athens, Greece; ^2^ University of California, San Francisco, San Francisco, CA, United States; ^3^ School of Medicine, University of Patras, Patras, Greece

**Keywords:** bone marrow failure, myelodysplastic neoplasms (MDS), targeted therapy, PNH (paroxysmal nocturnal haemoglobinuria), HLH (hemophagocytic lymphohistiocytosis), SF3B1 gene mutation

Inherited and acquired bone marrow failure syndromes (BMFS) represent a diverse group of diseases that share common pathophysiologic features. The rarity, the complex pathophysiology, and the protean clinical features of BMFS make diagnosis challenging, while treatment approaches are in many cases ineffective (1).

In this special research section entitled “*Biological Aspects of Bone Marrow Failure*” eight articles focusing on the pathophysiology of BMFS have been published.

In the article by Gavriilaki et al., real-world data from aplastic anemia and paroxysmal nocturnal hemoglobinuria (PNH) patients from two centres from Northern Greece are discussed. Half of the adult patients described in the manuscript had undergone allogeneic hematopoietic stem cell transplantation with a 10-year survival rate of approximately 57%. Based on the data available in the literature, regarding the upfront use of eltrombopag, transplantation can be reserved only for children and adults who do not respond to the combination of immunosuppressive treatment and eltrombopag.

In the article by Paolino et al., the authors describe the complex entity of hemophagocytic lymphohistiocytosis (HLH). The authors elegantly describe how HLH can be diagnosed and emphasize on the differences between primary and secondary HLH. The pathophysiology of HLH suggests that pro-inflammatory cytokines are mainly responsible for bone marrow suppression and subsequent cytopenias in the peripheral blood in at least two lineages. The authors underscore that HLH should be considered in every patient with bone marrow failure of unclear etiology.

In the article by Chatzileontiadou et al., the characterization and management of thromboembolic events in PNH patients are portrayed. PNH represents a highly thrombophilic disorder, with thrombosis being the most common and life-threatening complication in those patients. Moreover, the management of thromboembolic events can be challenging in daily clinical practice. In this article the authors highlight the fact that despite early initiation of complement inhibitors, at least one third of PNH patients will suffer a thrombosis. The size of the PNH clone and lactate dehydrogenase levels correlate with the incidence of thrombosis. Thromboses in unusual sites are quite common in PNH; furthermore, abdominal discomfort with hepatic dysfunction and splenomegaly are more frequent in patients with thrombosis.

In the article by Roka et al., the telomere biology and related diseases are discussed. The “telomeropathies” are diverse and there is a link between telomere length and disease outcome. Short telomeres have been associated with an increased risk of degenerative diseases like coronary artery disease, interstitial lung disease, and type I diabetes. The authors describe different diseases associated with short telomeres and their distinct clinical features. On the other hand, long telomeres result in the maintenance of genomic instability and have been associated with certain types of cancer, mainly melanoma and glioma. The authors discuss in detail the association of telomeres with BMFS (mainly dyskeratosis congenita and aplastic anemia) and acute and chronic leukemias. Understanding telomeres and telomerase activity can lead to the development of drugs targeting these mechanisms.

In the article by Fiesco-Roa et al., the authors discuss the pathophysiology of inherited bone marrow failure syndromes (IBMFS) focusing on Fanconi anemia and dyskeratosis congenita/telomere biology disorders. In this review, the disrupted cellular processes leading to the phenotypic features of the contrasting phenotypes of those disorders are analyzed. The authors, through their meticulous analysis, provide useful information that may permit the prompt identification of those patients, so that the correct diagnosis can be established, even before the onset of hematological or oncological manifestations.

In their review article, Pontikoglou et al. summarize existing findings on the aberrations within the bone marrow microenvironment that contribute to myelodysplastic neoplasm (MDS) initiation and evolution. This interesting article focuses on a less studied aspect of the pathogenesis of MDS apart from the genetic and epigenetic changes that are in the spotlight during the last few decades. The role of the bone marrow mesenchymal cells in the pathogenesis of MDS is highlighted through the description of a large number of studies supporting the assumption that those cells play a major role in the hematopoietic failure characterizing MDS.

On the other hand, Jiang et al. review data on the most frequent splicing factor mutation in MDS, that of splicing factor 3B subunit 1A (SF3B1). Given the fact that MDS with SF3B1 mutations have been classified as a separate subtype in the latest World Health Organization classification for MDS, and since SF3B1 mutations have been shown to play several distinct roles in the clinical features, diagnosis, prognosis, and therapeutics of this MDS subcategory, this interesting review article elaborates on the role of SF3B1 mutations as a potential therapeutic target possibly modifying the disease process.

Finally, the genetics of MDS, seen as an opportunity for tailored treatments, is the subject of a review article by Kontandreopoulou et al.. The authors thoroughly analyze the mutational landscape of MDS, providing a methodically organized description of somatic mutations of the RNA-splicing machinery, epigenetic mutations, signal transduction and transcription factors mutations, as well as mutation of the cohesin complex and the DNA repair genes, and finally the role of immune checkpoints. For each one of these genetic aberrations, the authors discuss the physiologic role of the implicated genes, the biological effect of the mutations, the available preclinical and clinical data on their significance, and finally the opportunities for tailored targeted treatments.

Through this Research Topic, we give to the reader the opportunity to focus on several aspects of the biology of BMFS, with original and review articles that provide new and noteworthy data on BMFS and MDS.

## Author contributions

PD: Conceptualization, Writing – original draft, Writing – review & editing. SA: Writing – original draft. ES: Conceptualization, Writing – original draft, Writing – review & editing.
